# Supplementary material for: Clinical significance of small nuclear ribonucleoprotein U1 subunit 70 in patients with hepatocellular carcinoma
Source: PeerJ. 2024 Mar 15;12:e16876. doi: 10.7717/peerj.16876 (PMC10946392; doi:10.7717/peerj.16876)
Supplement: Supplemental Information 1 [file peerj-12-16876-s001.docx]

**Supplementary Table 1 Sequenced of the three pairs of gRNAs designed online**

| gRNA code | Oligo name | Sequence |
| --- | --- | --- |
| gRNA-1 | Forward | CaccGTGACCGCGAGCACAAACGGGGG |
|  | Reverse | aaacCCCCCGTTTGTGCTCGCGGTCAC |
| gRNA-2 | Forward | CaccgTTACCTCTCATCGTAGCGGGAGG |
|  | Reverse | aaacCCTCCCGCTACGATGAGAGGTAAc |
| gRNA-3 | Forward | CaccgCGTGACCGCGAGCACAAACGGGG |
|  | Reverse | aaacCCCCGTTTGTGCTCGCGGTCACGc |
