# Supplementary material for: Clinical significance of small nuclear ribonucleoprotein U1 subunit 70 in patients with hepatocellular carcinoma
Source: PeerJ. 2024 Mar 15;12:e16876. doi: 10.7717/peerj.16876 (PMC10946392; doi:10.7717/peerj.16876)
Supplement: Supplemental Information 2 [file peerj-12-16876-s002.docx]

| **Supplementary Table 2**. Correlation between SNRNP70 expression and clinicopathological characteristics  in 278 patients with HCC. | | | | |
| --- | --- | --- | --- | --- |
|  |  | SNRNP70 |  |  |
| Variable | | Low | High | p |
| Sex |  |  |  | 0.143 |
|  | Male | 126 | 118 |  |
|  | Female | 13 | 21 |  |
| Age |  |  |  | 0.336 |
|  | < 50 | 61 | 69 |  |
|  | >50 | 78 | 70 |  |
| HBsAg |  |  |  | 0.866 |
|  | negative | 21 | 20 |  |
|  | positive | 118 | 119 |  |
| serum AFP | |  |  |  |
|  | < 20 ng/ml | 57 | 40 | 0.032 |
|  | >20ng/ml | 82 | 99 |  |
| Liver cirrhosis | |  |  | 0.371 |
|  | no | 49 | 42 |  |
|  | yes | 90 | 97 |  |
| TNM |  |  |  | 0.928 |
|  | I | 48 | 45 |  |
|  | II | 72 | 74 |  |
|  | III-IV | 19 | 20 |  |
| Child-pugh class | |  |  | 0.529 |
|  | A | 128 | 125 |  |
|  | B | 11 | 14 |  |
| Tumor size | |  |  | 0.23 |
|  | <5 cm | 72 | 62 |  |
|  | >5 cm | 67 | 77 |  |
| Tumor number | |  |  | 0.382 |
|  | single | 106 | 112 |  |
|  | multiple | 33 | 27 |  |
| tumor differentiation | |  |  | 0.98 |
|  | well | 14 | 13 |  |
|  | moderate | 124 | 125 |  |
|  | Poor | 1 | 1 |  |
| vascular invasion | |  |  | 0.712 |
|  | no | 55 | 52 |  |
|  | yes | 84 | 87 |  |
