# Supplementary material for: Clinical significance of small nuclear ribonucleoprotein U1 subunit 70 in patients with hepatocellular carcinoma
Source: PeerJ. 2024 Mar 15;12:e16876. doi: 10.7717/peerj.16876 (PMC10946392; doi:10.7717/peerj.16876)
Supplement: Supplemental Information 3 [file peerj-12-16876-s003.docx]

| **Supplementary Table 3. Univariate and multivariate analysis of OS and TTR-related factors in 278 patients with HCC.** | | | | | | | | | | | | | |
| --- | --- | --- | --- | --- | --- | --- | --- | --- | --- | --- | --- | --- | --- |
|  |  |  |  | OS | |  |  |  |  |  | TTR | |  |
|  |  |  |  |  |  |  |  |  |  |  |  |  |  |
|  |  |  |  | multivariate | |  |  |  |  |  | multivariate | |  |
| factors |  | univariate p | HR |  | 95% Cl | p |  | univariate p | | HR |  | 95% Cl | p |
| Sex: Male vs Female |  | 0.16 |  |  |  |  |  | 0.495 |  |  |  |  |  |
| age: ≤ 50 vs > 50 |  | 0.753 |  |  |  |  |  | 0.609 |  |  |  |  |  |
| HBsAg: positive vs negative |  | 0.105 |  |  |  |  |  | 0.095 |  |  |  |  |  |
| serum AFP (ng/ml): ≤ 20 vs > 20 |  | <0.0001 |  |  |  |  |  | 0.001 |  | 1.43 |  | 1.010-2.027 | 0.044 |
| liver cirrhosis: yes vs no |  | 0.015 | 1.699 |  | 1.141-2.531 | 0.001 |  | 0.002 |  | 1.781 |  | 1.245-2.550 | 0.002 |
| TNM: I vs II vs III-IV |  | <0.0001 |  |  |  |  |  | <0.0001 |  |  |  |  |  |
| Child-pugh: A vs B |  | 0.364 |  |  |  |  |  | 0.32 |  |  |  |  |  |
| Tumor size: ≤ 5 vs > 5 |  | <0.0001 | 2.504 |  | 1.716-3.655 | <0.0001 |  | <0.0001 |  | 1.931 |  | 1.397-2.670 | <0.0001 |
| Tumor number:single vs multiple |  | <0.0001 | 1.894 |  | 1.305-2.749 | 0.001 |  | <0.0001 |  | 2.103 |  | 1.493-2.963 | <0.0001 |
| tumor differentiation:well vs moderate vs poor |  | 0.048 |  |  |  |  |  | 0.021 |  |  |  |  |  |
| vascular invasion: no vs yes |  | 0.005 |  |  |  |  |  | 0.007 |  |  |  |  |  |
| nuclear SNRNP70: low vs High |  | 0.038 |  |  |  |  |  | 0.192 |  |  |  |  |  |
| nuclear SNRNP70/AFP combination: all low vs at least one high vs all high |  | <0.0001 | 1.456 |  | 1.132-1.874 | 0.003 |  |  |  |  |  |  |  |
