# Supplementary material for: Clinical significance of small nuclear ribonucleoprotein U1 subunit 70 in patients with hepatocellular carcinoma
Source: PeerJ. 2024 Mar 15;12:e16876. doi: 10.7717/peerj.16876 (PMC10946392; doi:10.7717/peerj.16876)
Supplement: Supplemental Information 4 [file peerj-12-16876-s004.docx]

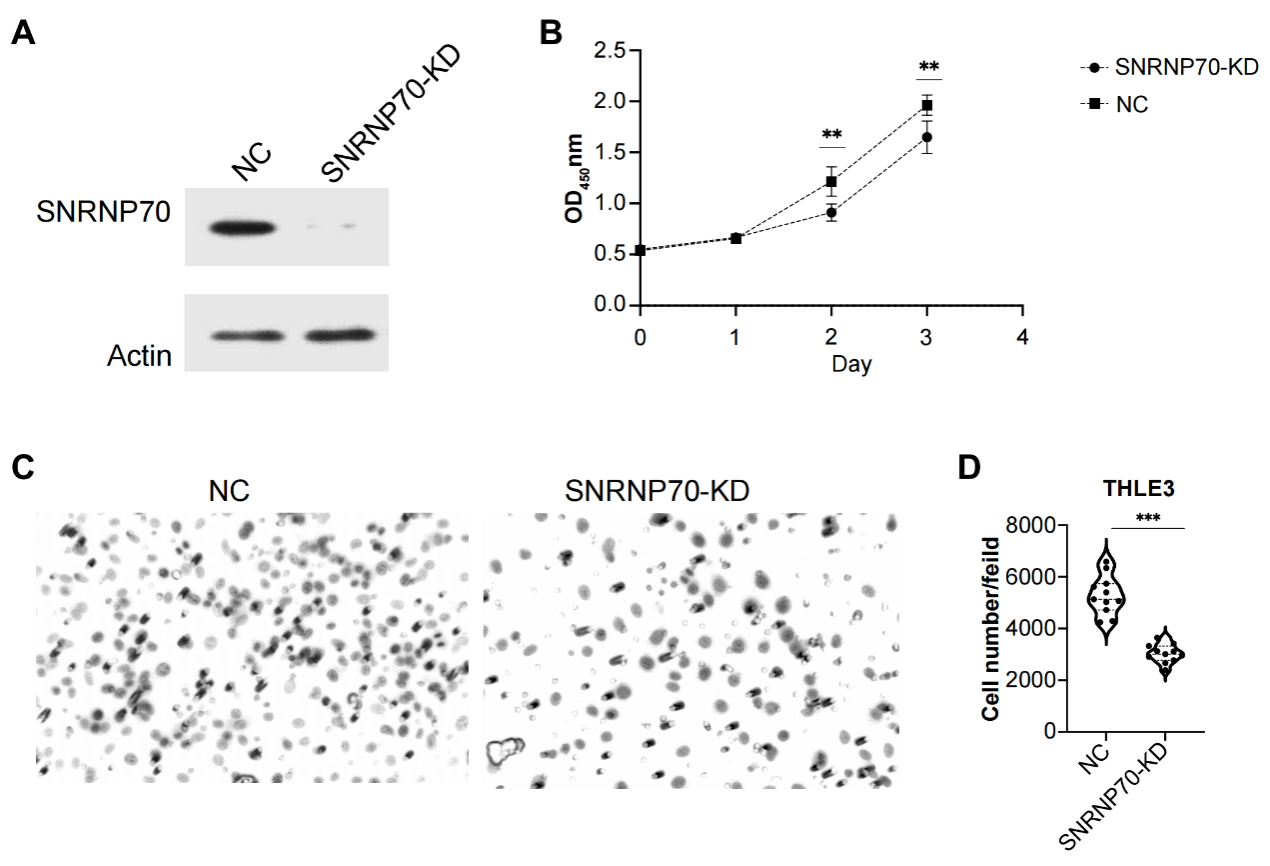


**Supplementary Figure 1.**  **SNRNP70 inhibits the proliferation and migration of THCE3 cells.**

(A) The protein expression level after the downregulated of SNRNP70 in THCE3 cells was detected by WB. (B) Down-regulated SNRNP70 in CCK-8 experiments inhibited the proliferation of THCE3 cells. (C-D) The effect of down-regulation of SNRNP70 on THCE3 cells migration was measured by the scratch wound healing assay. ***P*<0.01, ****P*<0.001.
